# Male-mediated species recognition among African weakly electric fishes

**DOI:** 10.1098/rsos.170443

**Published:** 2018-02-14

**Authors:** Rebecca Nagel, Frank Kirschbaum, Jacob Engelmann, Volker Hofmann, Felix Pawelzik, Ralph Tiedemann

**Affiliations:** 1 Institute of Biochemistry and Biology, Unit of Evolutionary Biology/Systematic Zoology, University of Potsdam, 14476 Potsdam, Germany; 2 Faculty of Life Sciences, Albrecht Daniel Thaer-Institute of Agricultural and Horticultural Sciences, Unit of Biology and Ecology of Fishes, Humboldt University of Berlin, 10115 Berlin, Germany; 3 Active Sensing, Faculty of Biology, Cognitive Interaction Technology – Center of Excellence, Bielefeld University, 33602 Bielefeld, Germany

**Keywords:** *Campylomormyrus*, communication, electric fishes, pre-zygotic isolation, species recognition

## Abstract

Effective communication among sympatric species is often instrumental for behavioural isolation, where the failure to successfully discriminate between potential mates could lead to less fit hybrid offspring. Discrimination between con- and heterospecifics tends to occur more often in the sex that invests more in offspring production, i.e. females, but males may also mediate reproductive isolation. In this study, we show that among two *Campylomormyrus* African weakly electric fish species, males preferentially associate with conspecific females during choice tests using live fish as stimuli, i.e. when all sensory modalities potentially used for communication were present. We then conducted playback experiments to determine whether the species-specific electric organ discharge (EOD) used for electrocommunication serves as the cue for this conspecific association preference. Interestingly, only *C. compressirostris* males associated significantly more with the conspecific EOD waveform when playback stimuli were provided, while no such association preference was observed in *C. tamandua* males. Given our results, the EOD appears to serve, in part, as a male-mediated pre-zygotic isolation mechanism among sympatric species. However, the failure of *C. tamandua* males to discriminate between con- and heterospecific playback discharges suggests that multiple modalities may be necessary for species recognition in some African weakly electric fish species.

## 1. Background

Many environmental and genetic components may influence the evolution and maintenance of reproductive isolation, including ecological specializations, behavioural mate discrimination and/or less fit hybrid offspring [1,2]. Among sympatric species, reproductive isolation may be further underscored by a given individual's ability to discriminate between con- and heterospecifics and by their strength of preference (SOP) for conspecifics [3]. However, males and females often differ in mate recognition and conspecific preference [4,5]. Reproductive isolation is thereby traditionally attributed to the sex that invests more in offspring production, i.e. females [6]. Male-mediated reproductive isolation has nonetheless been documented in several species, where mate recognition and assessment is facilitated by a wide range of sensory modalities [7–9]. The divergence of those communication cues responsible for species recognition and mate choice often leads to reproductive isolation and speciation [10–13].

African weakly electric fishes (Mormyridae) communicate by means of a species-specific electric organ discharge (EOD) generated with an electric organ [14–16]. Among certain mormyrid lineages, the diversification of this electric discharge has evolved more rapidly than body size, morphology and trophic ecology, suggesting that bioelectrogenesis was an important factor driving the speciation of electrogenic fishes [17]. This is exemplified by the African weakly electric fish genus *Campylomormyrus*. Endemic to the African freshwater river systems; all but one (*C. phantasticus*) of the 15 described *Campylomormyrus* species are found in sympatry in the Congo Basin [18]. Most of the external morphological differences between these species are confined to the trunk-like snout, which coincides with species-specific differences in the EOD waveform [18,19].

There is evidence that the EOD waveform functions in mate choice and species recognition, probably promoting pre-zygotic isolation among sympatric species [20–24]. Disruptive natural selection on characteristics associated with resource acquisition (snout morphology and EOD waveform) and reproductive isolation (EOD waveform) as well as the current sympatric occurrence of most species are consistent with sympatric speciation in this genus [18,25], though secondary syntopy cannot be ruled out with certainty.

Notwithstanding, discrimination based on the EOD waveform in some mormyrid genera appears to be asymmetric [26–29]. For example, female *C. compressirostris* discriminate between conspecific and *C. rhynchophorus* EOD waveforms, but do not discriminate between conspecific and *C. tamandua* EOD waveforms in mate choice tests using live fish and playback stimuli [26]. Given that the *C. rhynchophorus* EOD waveform is much longer, while the *C. tamandua* EOD waveform is more similar in duration to *C. compressirostris,* Feulner *et al*. [26] hypothesized that EOD duration played a key role in species recognition and discrimination. In this study, we present an alternative hypothesis, that mate choice and/or species recognition may be male-mediated. In doing so, we draw a distinction between mate choice and species recognition, in which both conspecific sexes are recognized as a unified category preferred over heterospecifics regardless of breeding status [30]. Furthermore, we investigate if the EOD waveform alone is indeed sufficient for pre-zygotic reproductive isolation.

Focusing on the preference behaviour of two sympatric African weakly electric fish species, *C. compressirostris* and *C. tamandua*, we aimed to address (i) if males discriminate between con- and heterospecifics where females do not, suggesting male-mediated species recognition and (ii) if the EOD mediates this conspecific association preference both during the breeding season and after gonadal regression.

## 2. Methods

### 2.1. Study species and experimental set-up

**Figure 1.**
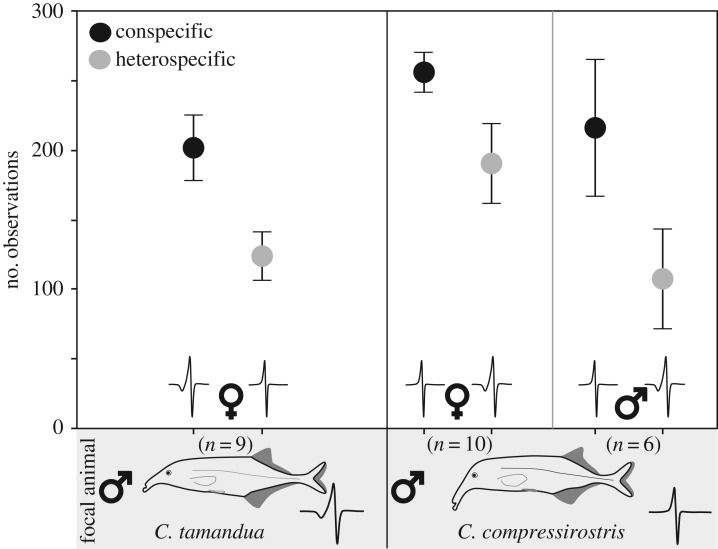
Choice tests with live fish as stimuli. Choice test results for *C. compressirostris* (*n* = 10) and *C. tamandua* (*n* = 9) males during interactions with live female and male con- and heterospecifics. EOD overlays represent the stereotypical waveform of each species. Association behaviour of male focal fish to con- and heterospecific stimuli is displayed as the mean (+s.e.) number of observations per night.

*Campylomormyrus compressirostris* (*n* *=* 17) and *C. tamandua* (*n* *=* 12) individuals were used in this study. The biphasic *C. compressirostris* EOD starts with a short, head-positive phase that is followed by a second head-negative phase with an average total duration of 164 µs. The triphasic EOD of *C. tamandua* has an initial small head-negative pre-phase followed by a head-positive and final head-negative phase; the average total duration is 316 µs (see EOD inlays, figure 1) [31]. Previous studies have demonstrated the uniformity of the EOD within single *Campylomormyrus* species, such that the EOD from any given individual is representative of the EOD produced by the species as a whole (e.g. [18,19]). As shown in other studies, neither under breeding conditions nor after gonadal regression was the EOD waveform sexually dimorphic in either species [31,32].

Prior to experiments, fish were kept in communal tanks under 12 L : 12 D cycle and fed daily with red and white mosquito larvae. Focal and stimuli fish came from different aquaria and were thus unfamiliar with each other and the test aquarium. Choice tests were conducted in a test aquarium (160 × 50 × 50 cm) partitioned into three compartments using plastic grids as separators (grid size: 13 × 13 × 13 mm; see electronic supplementary material). The focal fish was placed in the centre compartment (96 × 50 × 50 cm) 4 h prior to testing. Stimuli were located in two identical compartments (32 × 50 × 50 cm) on each side of the centre compartment (one live fish or dipole carbon electrode (for EOD playback) per compartment). The grid partitions separating the focal and stimuli fish allowed visual, olfactory and electric communication but prevented the fish from swimming freely throughout the tank. Two areas of 32 cm adjacent to each stimulus were marked on the exterior of the test aquarium and designated as ‘preference zones’ (see the electronic supplementary material). A plastic tube for shelter was provided in the centre of each compartment.

Breeding conditions were simulated by slowly decreasing water conductivity [32–34]. Fish were then held at a water conductivity of 250 ± 10 µS cm^−1^ and a temperature of 26 ± 2°C. With the exception of two individuals, all females (*C. compressirostris n* *=* 7; *C. tamandua n* *=* 3) laid eggs at least once under breeding conditions. All males showed a kink in their anal fin base indicating sex and maturity [26]. Concluding breeding condition experiments, the water conductivity was slowly increased to 700 ± 20 µS cm^−1^ to induce gonadal regression [32,34].

### 2.2. Experimental design

During choice tests using live fish as stimuli, male *C. compressirostris* (*n* *=* 10) and *C. tamandua* (*n* *=* 9) were presented with con- and heterospecific females (*C. compressirostris n* *=* 7 or *C. tamandua n* *=* 3). During a single test, one focal male was given the choice between one con- and one heterospecific female.

To test whether both conspecific sexes are recognized as a unified category preferred over heterospecifics, we then repeated the previous set-up using only male fish; *C. compressirostris* (*n* *=* 6) males were presented the choice between con- and heterospecific male individuals (*C. compressirostris n* *=* 5 or *C. tamandua n* *=* 6). For this test, we used any individual male either as a focal or as a stimulus fish. Owing to the small number of *C. tamandua* males (only nine specimens), this would have reduced our sample size to *n* = 4 (if *C. tamandua* had also been used as focal males). Therefore, this test was only performed with *C. compressirostris* as focal males.

Both *C. compressirostris* and *C. tamandua* are nocturnal. Male behaviour was therefore recorded for 12 h overnight (Microsoft LifeCam HD-3000; Spike2 video acquisitions, CED) under infrared illumination (880 nm). The video camera for recording was centred approximately 60 cm in front of the test aquarium. All preference tests were repeated with stimuli fish switched between sides to rule out side-bias, which entailed two nights of observations per individual. The overnight recordings were analysed using a custom written Matlab routine (R2015a, MathWorks Inc., Natick, MA, USA), where the focal fish's physical location in the test aquarium (i.e. in a pre-defined preference zone or in the neutral zone; see the electronic supplementary material, figure S1) was scored every 2 min, as in [26].

To test whether the species-specific EOD alone was sufficient to trigger a preference response, the previous set-up was repeated using digitally synthesized playbacks (see the electronic supplementary material). This was done once under breeding conditions and again after five months with the same individuals under non-breeding conditions (i.e. after gonadal regression) to further test if the association behaviour observed was linked to breeding behaviour. During playback, all possible sources of discrimination except EOD waveform were kept constant, including the sequence of pulse intervals (SPI), or temporal pattern of inter-EOD intervals. In contrast to the species-specific EOD, the SPI is variable and modulated by the fish depending on behavioural context (for review, see [35]). Little to no information is available about the function of inter-EOD intervals in socially isolated *Campylomormyrus* individuals, but natural SPIs have been shown to elicit stronger responses in mormyrids than artificial or scrambled sequences [36]. Additionally, a previous study reported that *C. tamandua* does not discriminate between the SPIs of con- and heterospecifics [24]. The inter-EOD-interval sequence selected for playback was therefore based on the SPI recorded from a randomly selected *C. compressirostris* female. We opted to use the same natural SPI in all playbacks to minimize any possible experimental variation/artefacts and to test only the influence of the EOD waveform on association preference. This experimental set-up is in accordance with other behavioural studies on African weakly electric fishes [22,26,29].

During choice tests using playback stimuli, male *C. compressirostris* (*n* = 10) and *C. tamandua* (*n* = 9) were presented with eight episodes of a 2 min SPI sequence, with a 2 min pause between two subsequent episodes (total playback time of 16 min) [23,26,29]. The orientation of the playback waveform (left versus right) was randomly interchanged after a given episode to control for any side-bias. Recording procedures (i.e. the position of video camera, recording equipment, software, etc.) were the same as in the choice tests using live fish as stimuli. The total time each focal male spent in the pre-defined preference zones was analysed using a custom-written Matlab routine.

The same individuals were used in all experiments, but there was a minimum of 30 days between the choice tests using live fish as stimuli and choice tests using playback stimuli. We also note that while mormyrids can be trained to distinguish minimal differences in individual EOD waveforms [21,22], no reward or punishment was associated with these experiments. The fish, therefore, were and remained untrained.

Experimental set-up and design were purposefully modelled after [26]. All raw data for our experiments are available in Dryad [37].

### 2.3. Statistics

We scored responses of *C. compressirostris* and *C. tamandua* males as the number of observations or total time spent (before and after switching side-assignments) associating with a given stimulus fish or playback electrode within the pre-defined preference zones, respectively. Previous experiments with other fish species, including mosquitofish [38], the redband darter [8] and gobies [39,40], suggest that position and time spent close to potential mates is a good proxy for preference respective mate choice; to the best of our knowledge, no information in this regard is available for mormyrids.

Data were analysed according to [41]. Shortly, the SOP was calculated as ((time spent near conspecific fish or playback stimulus – time spent near heterospecific fish or playback stimulus)/(time spent near conspecific fish or playback stimulus + time spent near heterospecific fish or playback stimulus)), so SOP values could range from −1 (complete avoidance of the conspecific fish or playback stimulus) to 1 (complete preference for conspecific fish or playback stimulus). SOP values were then tested against a random distribution of SOP = 0 using a one-sample *t*-test. A repeated measures ANOVA was used to compare the SOP of focal fish to playback stimuli during breeding and non-breeding conditions (i.e. after gonadal regression) using the *aov* function in the built-in R ‘stats’ package [42]. As the same individuals were tested in both conditions, both individual and reproductive stages were included as factors. Significant differences were evaluated with a post hoc Tukey's HSD pairwise comparison with a confidence interval of 95% using the R add-on ‘multcomp’ package [43]. We compared the SOP values of male *C. compressirostris* to male or female stimuli using a two-tailed *t*-test. All statistical tests were performed in R using the integrated development environment RStudio [42,44].

### 2.4. Research ethics and animal treatment

All specimens used in the experiment were imported from Kinshasa (Democratic Republic of the Congo) and are currently maintained at the University of Potsdam in Germany. All experiments were approved by the Deputy for Animal Welfare of the University of Potsdam and are in accordance with national legal requirements.

## 3. Results

To investigate the potential role of male species recognition and mate choice in pre-zygotic reproductive isolation between *C. compressirostris* and *C. tamandua*, we conducted dichotomous choice tests. When male focal fish were given the choice between live con- and heterospecific females, both *C. tamandua* and *C. compressirostris* males showed a significant association preference for conspecific females (one-sample *t*-test; *C. tamandua*: *t* = 2.725, d.f. = 8, *p* = 0.013 and *C. compressirostris*: *t* = 2.235, d.f. = 9, *p* = 0.026; figure 1). When male *C. compressirostris* focal fish were given the choice between live con- and heterospecific males, *C. compressirostris* males associated significantly more with conspecific males (one-sample *t*-test; *t* = 3.103, d.f. = 5, *p* = 0.013; figure 1). There was no significant difference in the SOP of *C. compressirostris* males to either conspecific females or males (two-sided *t*-test; *t* = 1.031, d.f. = 5, *p*-value = 0.35; figure 1).

**Figure 2.**
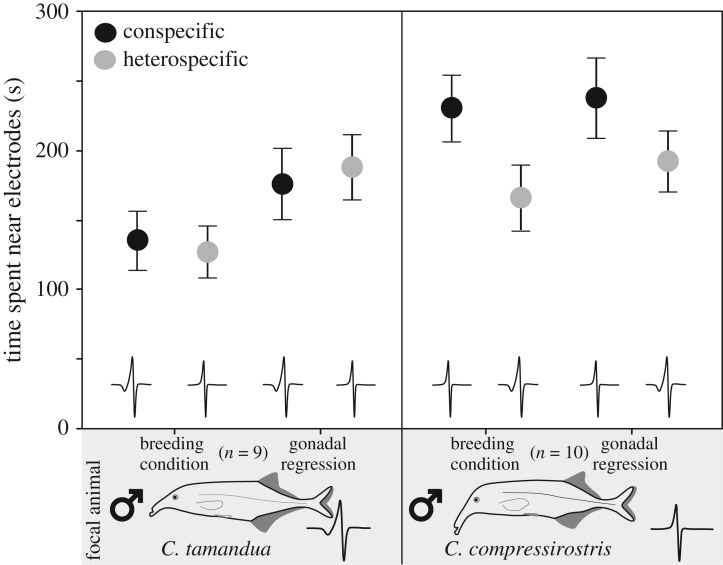
Choice tests with playback stimuli. Choice test results for *C. compressirostris* (*n* = 10) and *C. tamandua* (*n* = 9) males during interactions with playback stimuli, conducted during the breeding season and after gonadal regression. EOD overlays represent the stereotypical waveform of each species. Association behaviour of male focal fish to con- and heterospecific playback stimuli is displayed as the mean (+s.e.) time in seconds spent near the electrode per night.

To further test whether the species-specific EOD waveform was sufficient to elicit differential responses, we tested the preference behaviour of focal males using playback stimuli. Under breeding conditions, only *C. compressirostris* males associated significantly more with the conspecific playback (one-sample *t*-test; *t* = 4.021, d.f. = 9, *p* = 0.002). *Campylomormyrus tamandua* males did not discriminate between the two playback stimuli (one-sample *t*-test; *t* = 0.200, d.f. = 8, *p* = 0.423; figure 2). Also under non-breeding conditions (i.e. after gonadal regression), *C. compressirostris* males associated significantly more with the conspecific playback, whereas *C. tamandua* males did not (one-sample *t*-test; *C. compressirostris*: *t* = 5.948, d.f. = 9, *p* < 0.001 and *C. tamandua*: *t* = 0.814, d.f. = 8, *p* = 0.782). We found no significant difference in the SOP between the two trials (i.e. breeding conditions versus gonadal regression) for either *C. compressirostris* or *C. tamandua* males (analysis of variance, *p* = 0.160 and *p* = 0.558, respectively; figure 2).

## 4. Discussion

In this study, we investigated the association preference of two sympatric mormyrid weakly electric fish species, *Campylomormyrus compressirostris* and *C. tamandua*, and whether or not the EOD waveform facilitates species recognition under breeding and non-breeding conditions.

We show that in both species, males preferentially associate with con- versus heterospecific females in choice tests under breeding conditions using live fish as stimuli, i.e. with all sensory modalities available. Previous research has shown that female *C. compressirostris* do not discriminate between conspecific and *C. tamandua* males under comparable conditions [26], suggesting that males may be responsible for species recognition and discrimination. This corresponds with a recent hybrid breeding experiment, where *C. compressirostris* males and *C. tamandua* females (and vice versa) were held together in breeding groups; while females spawned eggs several times despite the absence of conspecific males, the eggs were never fertilized by the heterospecific males [45].

Interestingly, this preferential association with conspecifics was not only observed during male–female interactions. Male *C. compressirostris* also discriminated between male con- and heterospecifics, displaying a significantly stronger association preference for male conspecifics.

In previous studies with mormyrids, the ability to discriminate between con- and heterospecifics has frequently been associated with mate choice. However, a distinction should be made between mate choice and species recognition, another important aspect of behavioural isolation [30]. Given that *C. compressirostris* focal males respond equally to female and male stimuli, we suggest that the conspecific association preference observed may be male-mediated species recognition (i.e. a general attraction to conspecifics regardless of sex) rather than mate selection. Indiscriminate conspecific association preferences may have arisen as a social or shoaling behaviour, e.g. group foraging, which has been observed in some mormyrid species (*Mormyrops anguilloides* [46], *Marcusenius altisambesi* and *Mormyrus rume* [47]).

The communication cues facilitating species recognition are often fixed and unique to a species, and necessary for attracting a mate [30]. Among *Campylomormyrus* weakly electric fishes, the EOD is a species-specific but not sexually dimorphic component of electrocommunication [31,32]. We were therefore interested in investigating if the EOD waveform is necessary and sufficient for conspecific association and potentially mate recognition under both breeding and non-breeding (i.e. after gonadal regression) conditions. To do so, we repeated our choice tests with electrical playback stimuli, keeping all possible discrimination cues except the EOD waveform constant (e.g. EOD amplitude, inter-EOD-interval sequence, and electrode position and orientation in the tank). Under the hypothesis that the EOD waveform is sufficient to elicit a differential response, we predicted that both *C. compressirostris* and *C. tamandua* males would preferentially associate with the playback sequence pulse coupled with their own species' EOD waveform. While this prediction was supported for *C. compressirostris* males, *C. tamandua* males did not show the expected preference. This was true regardless of the conditions under which the experiment was conducted (i.e. under simulated breeding conditions or after induced gonadal regression). While the absence of an association preference does not inherently imply a lack of perceptual discrimination, these results may suggest that in the absence of other cues, the EOD waveform provides sufficient information for species recognition in some but not all African weakly electric fishes. This should not, however, be interpreted to mean that the electric modality is not at all relevant for recognition in these fishes.

In addition to the species-specific EOD waveform, species recognition in mormyrid fishes may be supported by various sensory modalities, including acoustic signals (*Pollimyrus* [48,49], *Brienomyrus* [50] and *Gnathonemus* [51]) and the lateral line or visual cues (*Gnathonemus* [52,53]). While little information is available about the function of olfactory systems or the production/detection of hormonal pheromones in mormyrids, pheromones are known to mediate species and mate recognition in numerous other teleost fishes [54]. Therefore, we do not exclude the possibility that chemoreception may also act synergistically with the electrosensory system. Indeed, the use of multiple signals for species recognition has been widely studied in various species [55]. Female swordtail fish *Xiphophorus pygmaeus* successfully discriminate between conspecific and sympatrically occurring heterospecific *X. cortezi* males only if presented with at least two species-specific cues, i.e. visual and chemical [56]. Among sympatric cricket species, female *Gryllodinus kerkennensis* identify conspecifics based solely on the continuity of the male song, while *Gryllus campestris* require both pulse rate and chirp duration for discrimination [57].

The temporal pattern with which the discharge is produced (sequence pulse interval, SPI) may also transmit important information for species recognition. Our study did not specifically focus on the importance of the temporal pattern of EODs for species recognition, but the inter-EOD interval has been linked to species identification (*Campylomormyrus* [24]), courtship and spawning behaviour (*Pollimyrus* [58]), and various other social interactions (*Gnathonemus* [36] and *Brienomyrus* [59]). Particular patterns of SPIs also appear to correlate with certain social contexts, as observed in the mormyrid genera *Marcusenius* and *Mormyrus* [47]. In one African weakly electric fish (*Pollimyrus isidori*), SPI cues alone were even sufficient for sex recognition and discrimination [48]. As all EOD waveforms were paired with a *C. compressirostris* SPI in our playback choice tests, we cannot rule out the possibility that *C. tamandua* males did not discriminate between con- and heterospecific EODs, because they were presented with a heterospecific SPI (i.e. from *C. compressirostris*). Nonetheless, a previous study found that *C. tamandua* could not or did not discriminate between con- and heterospecific SPIs [24].

Overall, our results indicate that in at least two African weakly electric fish species, males can discriminate between con- and heterospecific individuals when live fish are provided as stimuli, whereas a previous study demonstrated that females do not exhibit such discrimination [26]. This suggests that male behaviour may be an important component of pre-zygotic isolation and therefore a mechanism of speciation among these sympatric species. We further hypothesize that species recognition rather than mate selection is driving this conspecific association preference. It should be emphasized that both *C. compressirostris* and *C. tamandua* share an EOD of similar duration and shape, however with subtle differences in the pre-phase (i.e. regarding the occurrence of a small initial head-negative phase; see EOD inlays, figure 1). While these species-specific EOD waveform characteristics seem to be a strong cue for species recognition, our results show that it is not sufficient in all cases.

It is important to mention the limited sample size of this study, which is most notably restricted by access to only three *C. tamandua* females for choice tests with live fish as stimuli. The two *Campylomormyrus* species investigated also limit our results; by including more species, it would be possible to discern, for example, if male-mediated species recognition is prevalent in all 14 sympatrically occurring *Campylomormyrus* species. Future investigations will also need to clarify which species-specific cues from various other modalities might play a role in mediating successful species recognition.

## Supplementary Material

Supplementary material for “Male-mediated species recognition among African weakly electric fishes”

## Data accessibility

The data used in this study are available in the Dryad Digital Repository: (http://dx.doi.org/10.5061/dryad.9kv93) [37].
