# Supplementary material for: Male-mediated species recognition among African weakly electric fishes
Source: R Soc Open Sci. 2018 Feb 14;5(2):170443. doi: 10.1098/rsos.170443 (PMC5830707; doi:10.1098/rsos.170443)
Supplement: Supplementary material for “Male-mediated species recognition among African weakly electric fishes” [file rsos170443supp1.docx]

**Supplementary material**

Nagel R, Kirschbaum F, Engelmann J, Hofmann V, Pawelzik F, Tiedemann R. Male-mediated species recognition among African weakly electric fishes. *Royal Society Open Science*


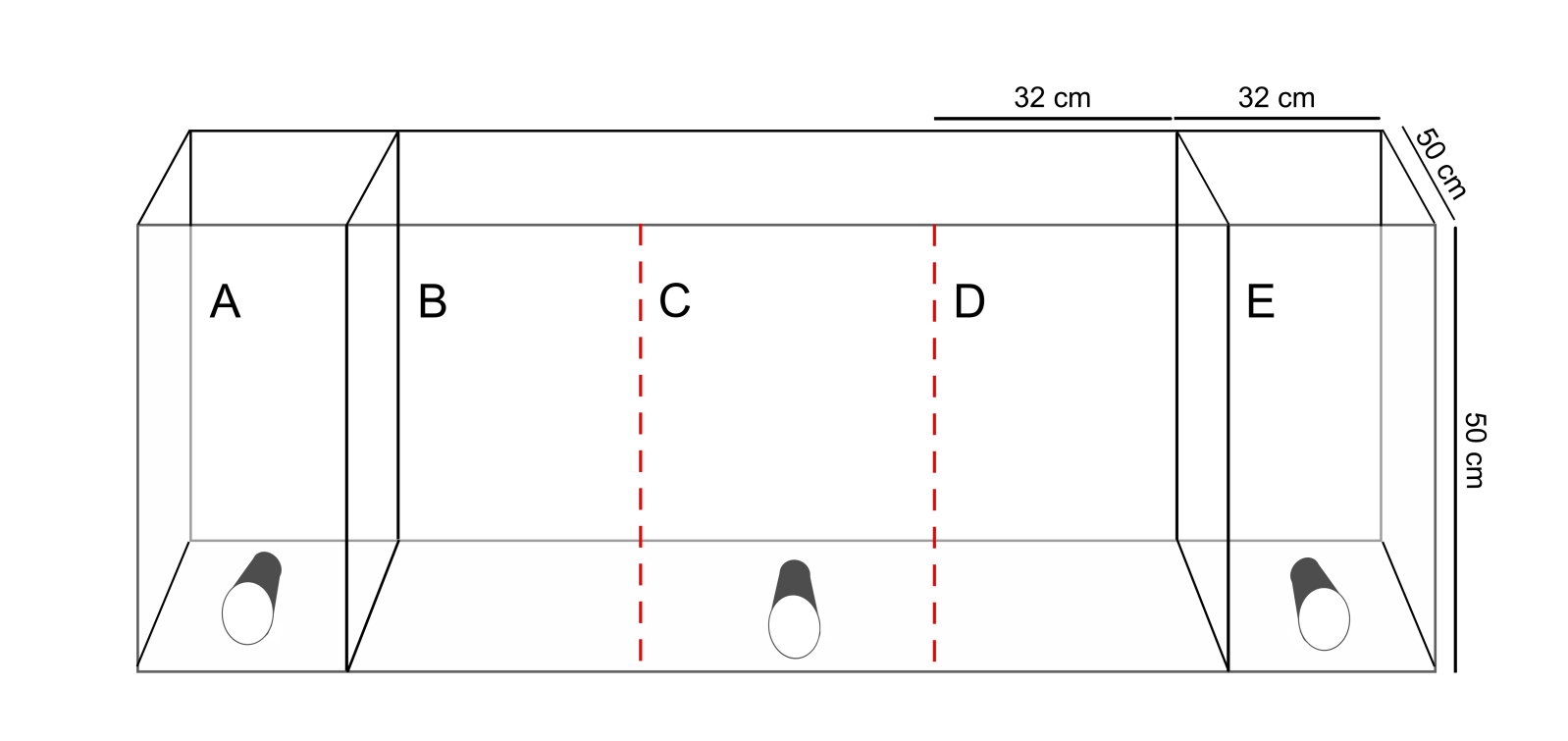
Figure S1. The test aquarium used for experiments was divided into five sections (marked A - E). A stimulus fish or playback electrode was located behind the gridded partitions in sections A or E. The focal fish was located in the centre of the aquarium, which was divided visually into three equal sections (marked B-D). Section B and D represent the preference zones for A and E, respectively, while section C was considered a neutral zone. A tube for shelter was provided in sections A, C, and E.


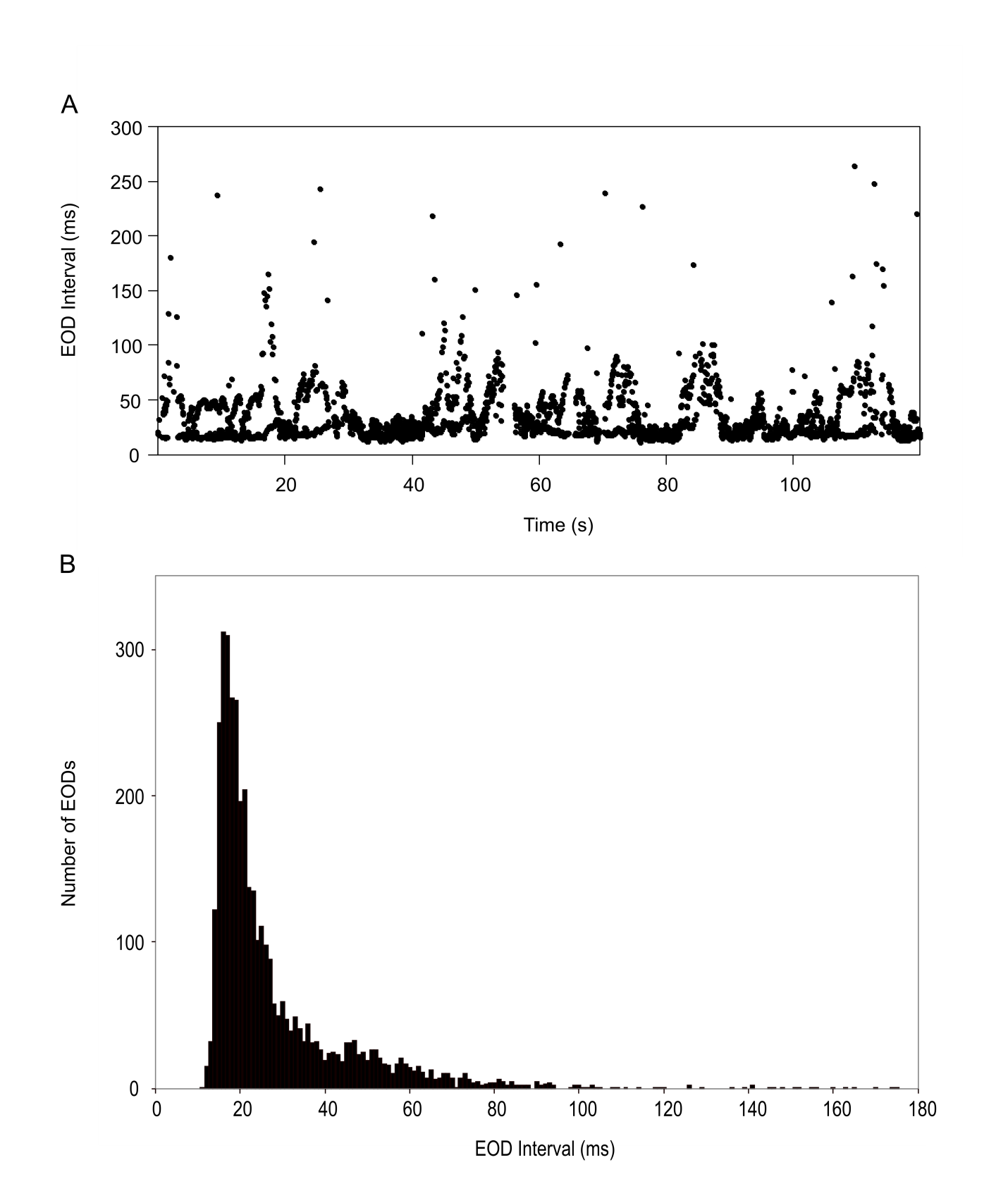


Figure S2. (A) Sequence pulse interval (SPI) of the playback stimuli used in choice tests. (B) Cumulative histogram showing the distribution of electric organ discharge (EOD) intervals for the 2-minute playback sequence.

Experimental Design: Recording EODs and SPI Sequences

To record a representative electric organ discharge (EOD) for each species, randomly selected females from *C. compressirostris* and *C. tamandua* were transferred into a recording aquarium (40 × 20 × 16 cm, water conductivity: 250 ± 5 μS/cm, temperature: 26°C). Opposing ends of the aquarium were fitted with fine silver wire electrodes, with the positive pole at the anterior of the fish. To prevent the fish from swimming freely during measurements, it was held in a plastic tube with plastic mesh on both ends and placed equidistance between the two electrodes. The EODs were amplified (MA 102; University of Cologne, Zoological Institute, Electronics Lab for Animal Physiology), digitized (CED MICRO 1401; Cambridge Electronic Design Limited), and visualized (Spike2, software; Cambridge Electronic Design Limited). Per individual, 60 seconds were recorded. Given that the EOD from any given individual is representative of the EOD produced by the species as a whole (e.g. [1,2]), one 60-second recording for each species was randomly selected to create an average EOD. This is in accordance with procedures reported by other groups (i.e. [3,4]). The average waveform for each species was created using Spike2 and MATLAB (R2015a, MathWorks Inc., Natick, MA USA) and uploaded to a Rigol DG1032Z waveform generator for later playback.

The stimulus train used for playback experiments was recorded from a randomly chosen, free-swimming *C. compressirostris* female at night (water conductivity: 259 μS/cm, temperature: 26°C; Fig. S2). We choose this sequence pulse interval (SPI) for our playback because we expected a natural SPI to elicit stronger responses than an artificial or scrambled rhythm [5]. Additionally, a previous study reported that *C. tamandua* did not discriminate between the SPI patterns of con- and heterospecifics [6]. One stimulus train was used throughout all choice tests with playback stimuli to ensure association preference was based only on variation in the EOD waveform; this is in accordance with procedures used in other studies (i.e. [3,4]). The stimulus train was recorded using Spike2 and MATLAB and analysed in R using the integrated development environment RStudio [7,8]. The resulting 2-minute sequence used during playback had 3858 inter-EOD-intervals with a median interval length of 17.8 ms (range: 11 – 1771 ms).

Experimental Design: Choice tests

Preference zone dimensions were determined during pilot experiments in the test aquarium, where isolated males (*n*  = 6) were recorded for 12 hours overnight. Isolated males spent, on average, over 85% of the time in the middle 32 cm of the aquarium in and around the plastic shelter. During choice tests, total time spent in the shelter area sometimes dropped as low as 23%, with the total average time spent in the shelter area across individuals just below 50%.

During playback experiments, the SPI sequence was output through the data acquisition unit (CED MICRO 1401), which triggered playback of the EOD waveform stored on the waveform generator (Rigol DG1032Z). The SPI sequence was coupled with the respective average EOD waveform (*C. compressirostris* or *C. tamandua*) in the Rigol DG1032Z, which then simultaneously output the coupled signals through two independently controlled channels, emitting the signals of the two species respectively on the two opposite sides of the test aquarium (Fig. S1). Given that the amplitude of the electric discharge is a function of the distance between the fish and the recording electrodes [5], the amplitude of each digital EOD was modified in the Rigol DG1032Z so that the playback amplitude matched that of a live fish at equidistance. The starting time of the SPI sequence was shuffled by 60 seconds so the two channels played the same total number of EODs each interval, but the output did not overlap. Each channel output was isolated in a Model 2200 Analog Stimulus Isolator (A-M Systems, Inc.). The signals were played into the tank through electrodes situated behind plastic grid partitions at opposite ends of the aquarium, 53 cm from the middle. Each electrode was oriented with positive and negative poles parallel to the long axis of the aquarium.

References

1. Feulner PGD, Kirschbaum F, Schugardt C, Ketmaier V, Tiedemann R. 2006 Electrophysiological and molecular genetic evidence for sympatrically occuring cryptic species in African weakly electric fishes (Teleostei: Mormyridae: Campylomormyrus). *Mol. Phylogenet. Evol.* **39**, 198–208. (doi:10.1016/j.ympev.2005.09.008)

2. Lamanna F, Kirschbaum F, Ernst ARR, Feulner PGD, Mamonekene V, Paul C, Tiedemann R. 2016 Species delimitation and phylogenetic relationships in a genus of African weakly-electric fishes (Osteoglossiformes, Mormyridae, Campylomormyrus). *Mol. Phylogenet. Evol.* **101**, 8–18. (doi:10.1016/j.ympev.2016.04.035)

3. Feulner PGD, Plath M, Engelmann J, Kirschbaum F, Tiedemann R. 2009 Electrifying love: electric fish use species-specific discharge for mate recognition. *Biol. Lett.* **5**, 225–228. (doi:10.1098/rsbl.2008.0566)

4. Arnegard ME, Jackson BS, Hopkins CD. 2006 Time-domain signal divergence and discrimination without receptor modification in sympatric morphs of electric fishes. *J. Exp. Biol.* **209**, 2182–2198. (doi:10.1242/jeb.02239)

5. Teyssedre C, Serrier J. 1986 Temporal spacing of signals in communication, studied in weakly-electric mormyrid fish (Teleostei, Pisces). *Behav. Processes* **12**, 77–98. (doi:10.1016/0376-6357(86)90073-2)

6. Kramer B, Kuhn B. 1994 Species recognition by the sequence of discharge intervals in weakly electric fishes of the genus Campylomormyrus (Mormyridae, Teleostei). *Anim. Behav.* **48**, 435–445. (doi:10.1006/anbe.1994.1257)

7. R Core Team. 2014 R: A language and environment for statistical computing.

8. RStudio Team. 2015 RStudio: Integrated Development for R.
